# The “respiratory REM sleep without atonia benefit” on coexisting REM sleep behavior disorder - obstructive sleep apnea

**DOI:** 10.5935/1984-0063.20200054

**Published:** 2021

**Authors:** Daniela L. Giardino, Paola Fasano, Arturo Garay

**Affiliations:** Centro de Educación Médica e Investigaciones Clínicas “Norberto Quirno” (CEMIC), Neurology- Sleep Medicine - Ciudad de Buenos Aires - Buenos Aires - Argentina.

**Keywords:** REM Sleep Parasomnias, REM Sleep Behavior Disorder, Obstructive Sleep Apnea, Obstructive

## Abstract

Rapid eye movement sleep behavior disorder (RBD) is a parasomnia characterized by dream-enactment behaviors that emerge during a loss of REM sleep atonia. In patients with RBD, obstructive sleep apneas syndrome (OSAS) frequently occurs as a comorbid entity. It has been reported that the presence of muscle tone during REM sleep (REM sleep without atonia-RSWA) could play a protective role in patients with OSAS RBD. In OSAS, recurrent episodes of complete or partial collapse of the upper airway occur during both, NREM and REM sleep. Particularly during sleep, the withdrawal of excitatory noradrenergic and serotoninergic inputs to the upper airway motor neurons deeply reduces the pharyngeal muscle activity, increasing the propensity for superior airway collapse. The present study compared for the first time the impact of OSAS in RBD patients with a subtype of OSAS patients with predominantly or isolated REM sleep-related OSAS (OSAS REM group) in the search of an adequate model to evaluate future therapeutic strategies. Our study found a significant lower nadir of oximetry values in OSAS RBD in comparison with the OSAS REM group. This reduction, that we called the “respiratory RSWA benefit”, is in accordance with the decrease of the nadir oximetry values observed in patients with Parkinson disease and OSAS with or without RBD. We suggest that the group of OSAS REM patients is a natural model to evaluate the respiratory protective role of RSWA in patients with coexisting RBD-OSAS and Parkinson’s disease.

## INTRODUCTION

Rapid eye movement sleep behavior disorder (RBD) is a parasomnia characterized by dream-enactment behaviors that emerge during a loss of REM sleep atonia^1^. Typically, patients diagnosed with RBD performed dream enactment that ranges in severity from benign hand gestures to violent thrashing, punching, kicking and/or falling out the bed during abnormal REM sleep and are at risk for sleep-related injury to themselves or their bedpartner^1^^-^^2^. The prevalence of RBD is estimated to be 0.5 to 1.0% of the general population^3^^-^^5^ with a strong male gender predilection (male to female ratio 9:1). Despite the mean age of disease onset is 45-65 years, symptoms typically begin in late adulthood and, in general, there is a 4-5 years elapse between onset and diagnosis^6^^,^^7^. RBD can be caused by prolonged treatment with antidepressant medications (serotonin reuptake inhibitors, serotonin-norepinephrine uptake inhibitors, MAO inhibitors, and tricyclics), beta-blockers, withdrawal (alcohol and barbiturate), or during obstructive sleep apnea syndrome (OSAS), narcoleptic patients and patients with central nervous system injuries (classically patients with pontine lesions related to vascular or demyelinating diseases).

The diagnosis of RBD requires a clinical history of repeated episodes of sleep related motor behaviors plus REM sleep without atonia (RSWA) captured with polysomnography^1^^,^^8^. The term idiopathic RBD (iRBD) refers to RBD occurring in the absence of any other neurological disorder or any other possible cause. A growing body of clinical studies have proposed that idiopathic RBD is a risk factor for the development of abnormal alpha-synuclein mediating neurodegenerative diseases (Parkinson’s disease - PD, multisystem atrophy - MSA or Lewy body disease - LBD), with an estimated rate of phenoconversion over a lifetime of 81 to 90%^9^. RBD is widespread among these patients with a high prevalence in PD, MSA and LBD^10^. A recent multicentric work that includes 1280 patients, found an overall conversion rate from idiopathic RBD to a neurodegenerative disorder of 6.3% per year with 73.5% converting after 12-years follow-up^7^. Thus, more than two decades of research in RBD have extensively demonstrated its importance as a sleep biological marker of alpha-synucleinopathies.

In patients with RBD, OSAS^11^^,^^12^ can occur frequently as a comorbid entity^13^. In OSAS, recurrent episodes of complete or partial collapse of the upper airway occur during both, NREM and REM sleep. During REM sleep, the withdrawal of excitatory noradrenergic and serotoninergic inputs to the upper airway motor neurons deeply reduces the pharyngeal muscle activity, increasing the propensity for superior airway collapse. It has been hypothesized that the presence of muscle tone during REM could play a protective role in patients with OSAS during REM sleep (OSAS REM)^14^^,^^15^. In contrast, other authors have suggested that in patients with Parkinson’s disease (PD) the complex of RBD-OSA presents a more profound respiratory alteration^16^. In addition, other reports found either no relation between chin muscle tone or the frequency of apneic events in PD^17^ or increased respiratory alteration in patient with another synucleinopathies (multiple system atropy - MSA and dementia of Lewy bodies - DLB during supine position) but not alteration in PD^18^. Taking into account this controversy we decided to study the respiratory parameters of patients with REM related OSA (OSAS REM), in comparison with patients presenting both OSAS and RBD (OSAS RBD). In OSAS REM patients the presence of obstructive events occurred predominantly or exclusively during REM sleep. It is considered that 10-36% of patients with obstructive sleep apnea suffered from REM-related OSA^19^.

Our hypothetical point of view was that the reduction of inhibitory motor control expressed during REM sleep muscle activity, in RBD patients, could have a protective role decreasing the expression of respiratory events comparing with the population of OSAS-REM patients.

## MATERIAL AND METHODS

We reviewed studies of patients who underwent nocturnal polysomnography (PSG) or video PSG (vPSG) at the Sleep Laboratory, Department of Neurology, Centro de Educación Médica e Investigaciones Clínicas Norberto Quirno (CEMIC), Buenos Aires, Argentina. During this retrospective case-control study a total of 51 participants were reviewed according to their clinic chart-PSGs/vPSGs. We evaluated 25 patients with iRBD and coexisting OSA (OSAS RBD) and 26 patients with REM-predominant OSA and REM-isolated OSA that we included in a group called OSA-REM.

All subjects underwent clinical interviews and completed conventional scales like the Pittsburgh sleep quality index (PSQI) and Epworth sleepiness scale (ESS). One-night polysomnography (PSG) or video-polysomnography (vPSG) were also performed with digital polysomnographs (Bioscience/Harmonie, Buenos Aires, Argentina and Neurovirtual, Fort Lauderdale, FL, USA), recording oculography, electroencephalography (at least six channels F3-A2, F4-A1, C4-A1, C3-A2, O1-A2, O2-A1), electrocardiography activities, electromyography (EMG) activity of the mentalis, right and left tibial muscles, nasal air ﬂow, thoracic and abdominal respiratory effort, oxygen saturation, microphone, and digital EEG-synchronized videography with infrared camera.

Diagnoses of RBD and OSA were made according to standard criteria (ICSD-3)1. All participants were ≥50 years and met inclusion and exclusion criteria. We excluded patients with cardiac disease, dementia, signs or symptoms of parkinsonian-plus disorders or any additional neurodegenerative diseases, as well as patients who use alcohol or drugs with inﬂuence on the autonomic nervous system. This study was reviewed and approved by the CEMIC ethics committee.

All the OSAS-RBD patients presented episodes of dream enactment with excessive muscle activity or RSWA with a history of dream enactment that we analyzed using AAMS criteria’s^20^. Tonic excessive muscular activity was assessed in 30s epochs and considered when submental EMG activity exceeded twice that of background activity for more than 50% of the epoch. Phasic excessive muscular activity was measured in 3s mini-epochs and deﬁned as sub-mental EMG activity bursts lasting 0.1 to 0.5s and exceeding four times that of the background.

Respiratory sleep patterns were studied in line with standard criteria: apnea was deﬁned as absence of airﬂow lasting 10s, and hypopnea as a reduction of 50% in the amplitude of airﬂow signal lasting 10s and accompanied by oxygen desaturation of 4% and/or arousal according to AASM recommendations. Apnea and hypopnea were further divided as obstructive, central or mixed according to the presence or absence of respiratory effort. Oxygen desaturation was deﬁned as a reduction in pulse oximetry oxygen saturation by more than 3%. We calculated the apnea-hypopnea index (AHI), as the total number of apneas and hypopneas per hour of sleep. AHI was calculated as the total number of apnea-hypopnea episodes per hour of sleep (total sleep, REM sleep, and NREM sleep, respectively). REM-predominant OSAS was defined as a doubling of AHI in REM sleep versus the NREM sleep (AHI-REM/AHI-NREM >2) and REM-isolated OSA was characterized by a doubling of AHI in REM sleep in addition to an AHI of less than 5/h in NREM sleep (AHI-REM/AHI-NREM >2 and AHI-NREM <5)^21^.

Sample size was calculated using T statistic for a proposed power of 80% and error alfa of 5%. The minimum size was 24 patients by group. Descriptive statistics were given as mean + standard deviation, normality of the data was calculated by Shapiro-Wilk Test (W: 0.5077, *p*<0.0001). According to these results, nonparametric tests (Mann-Whitney U) were used to analyze the results. The *p*-value<0.05 was considered statistically significant. GraphPad Prism 8 for Windows was used for graphs and statistical analyses (GraphPad Software, LLC).

## RESULTS

From a total of 51 patients, 26 were selected with predominantly OSAS REM (two of them presented isolated OSAS REM) and 27 with OSAS RBD. Table 1 shows the comparison between OSAS RBD and OSAS REM patients. There were no statistical differences between groups with regard to age and body mass index. The ratio male/female was higher in the group of OSAS RBD although without reaching statistically significant difference. When sleep architecture and sleep continuity parameters were analyzed in both groups, no significant differences of the following parameters: TTS, WASO, SE, N1, N2, N3, REM stages were found (Table 1).

**Table 1:** Polisomnography data of OSAS RBD and OSAS REM patients.

	OSAS REM (n=26)	OSAS RBD (n=27)	P value
Age (years)	59.6±12.3	66.0±2.9	0.0971
Gender (m/f)	w 18/8	21/6	0.547
BMI (kg/m2)	32.5±4.1	31.7±6.0	0.7219
TTS (min)	347.4±66.4	329.6±76.4	0.7531
WASO (min.)	72.3±6.9	81.7±12.3	0.1040
SE (%)	89.3±7.3	80.7±12.3	0.1667
N1 (min.)	13.7±9.1	18.9±6.2	0.1443
N1 (of TTS, %)	4.1±3.0	7.2±3.3	0.0777
N2 (min.)	205.5±47.6	170.4±25.2	0.1377
N2 (of TTS, %)	59.3±9.1	63.2±8.5	0.4894
N3 (min.)	68.2±47.4	63.2±41.4	0.7531
N3 (of TTS, %)	19.2±12.0	41.6±52.4	0.8513
REM (min.)	59.9±23.8	36.4±15.5	0.0777
REM (of TTS, %)	17.3±6.4	11.6±3.5	0.0777
AHI	15.4±16.5	20.6±18.8	0.2733
AHINREM	9.4±7.9	16.8±14.4	0.3681
AHIREM	30.7±27.1*	17.3±17.4	0.9654
Mean SpO2 (%)	92.8±2.5	92.7±3.1	0.6355
Nadir SpO2 (%)	73.1±7.7	79.6±8.8	0.0037*

PSGs variables are expressed as mean±SD. BMI (body mass index, kg/m2); TTS (Total Sleep Time, min.); WASO (Wake after sleep time, min.); SE (sleep efficiency); AHI (Apnea-Hipopnea Index/hour); AHI NREM (Apnea-Hipopneas Index/hour NREM); AHI REM (Apnea-Hipopnea Index/hour REM); Mean SpO2 (Mean Pulse Oximetry, %); Nadir SpO2 (Nadir Pulse Oximetry, %).

* p < 0.05, Two-tailed (Mann Whitney U test).

**Figure 1:**
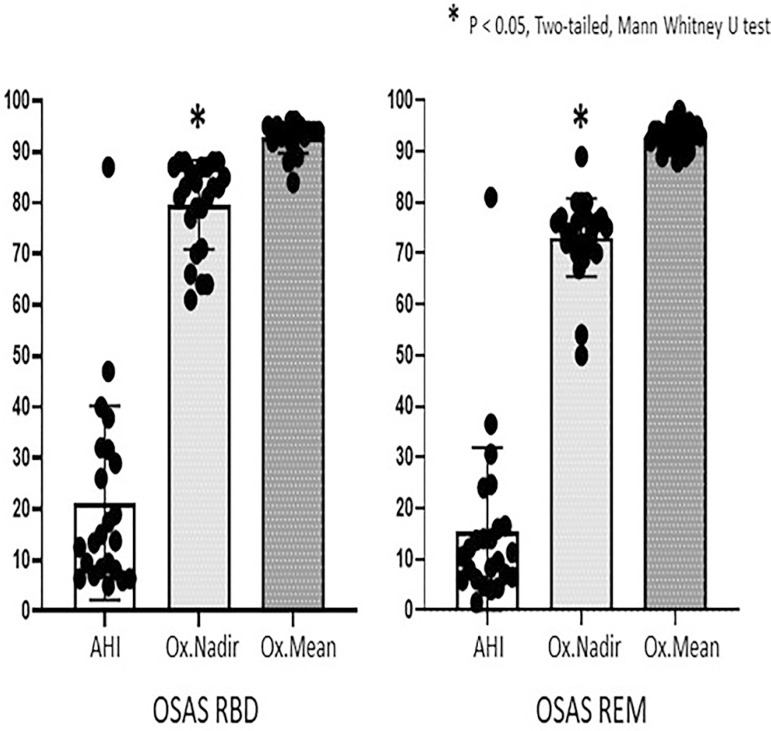
Comparison of respiratory-related parameters between OSAS RBD and OSAS REM patients. Ox.Mean of OSAS RBD and OSAS REM (Mean Pulse Oximetry, %); Ox. Nadir of OSAS RBD and OSAS REM (Nadir Pulse Oximetry, %). Ox.Nadir OSAS RBD vs Ox.Nadir OSAS REM (* p < 0.05, Two-tailed (Mann Whitney U test); AHI NREM OSAS REM vs. AHI OSAS REM (* p < 0.05, Two-tailed (Mann Whitney U test).

**Figure 2:**
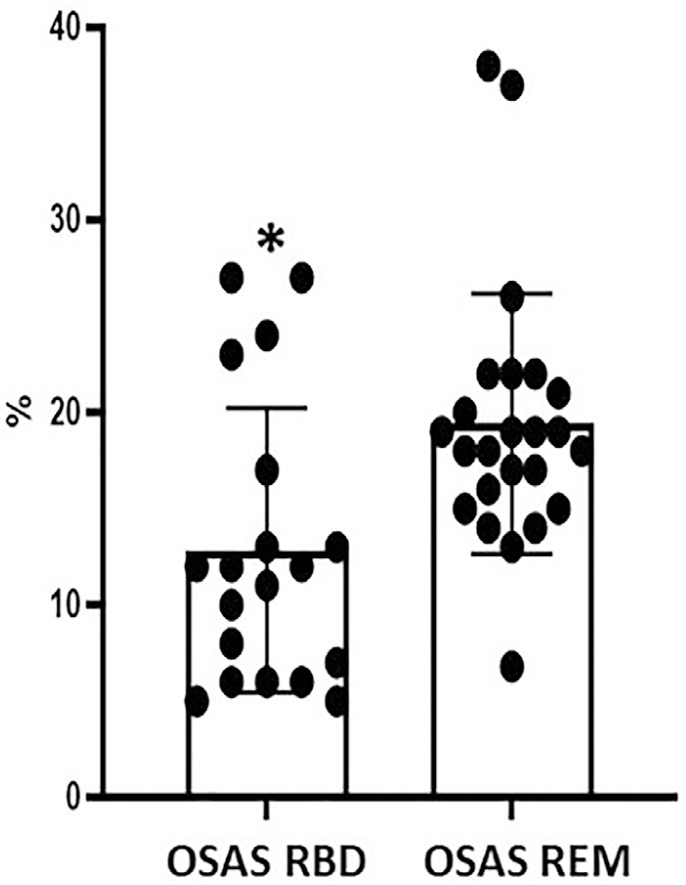
Percentage of the differences between Mean SpO2-Nadir SpO2 in OSAS RBD and OSAS REM patients. Percentage of drop in OSAS RBD (Mean±SD): 12.8±7.38; % of drop in OSAS REM (Mean±SD): 19.41±6.75; *p 0.0019, Two-tailed (Mann Whitney U test).

In relation with respiratory parameters, there were no significant differences in AHI, AHI NREM and AHI REM between groups, although differences between AHI NREM and AHI REM in the OSAS REM group (*p*<0.042) were evidenced. Mean SpO_2_ was similar between groups but the OSAS REM group achieved a significant lower nadir SpO_2_ compared to the OSAS RBD group (*p*<0.0037) (Figure 1 and Table 1). Figure 2 shows a significant lower nadir SpO_2_ in the OSAS REM group (19.41±6.75) compared to the OSAS RBD group (12.8±7.38, *p*<0.0019) when data were expressed as mean±SD of the percentage values of the difference of the individual mean SpO_2_ and nadir SpO_2_ of each group. This difference represents a lower drop of the nadir SpO_2_ of approximately 6.6% in OSAS RBD group with respect to OSAS REM group.

## DISCUSSION

Despite the overwhelming research related to RBD and OSAS as different entities, few papers pay attention to the coexistence of RBD and OSA. This coexistence is important, for at least two situations, the first one related to the presentation of pseudo RBD patients in whom the identification of sleep apnea and their treatment ameliorates or “treats” RBD and, the second, related to the evidence, still controversial, that suggest a protective effect on respiratory variables in RBD.

This finding has been proposed by Schenck et al., in 1992, given the increase in activity of EMG during REM sleep in patients with RBD (cited by Huang^14^). The pathophysiology of the mechanisms that lead to this increased activity are not clearly elucidated. However, it is accepted that anatomy of the airway is not further impaired in REM sleep than in NREM sleep and that more profound oxygen desaturation during REM sleep could be reduced, improving the genioglossus muscle tone^22^. Nevertheless, it remains unclear how excessive EMG activities may modulate the severity of OSA in RBD patients^14^^,^^23^.

We specifically evaluated polysomnographic patterns in patients with coexisting OSAS RBD comparing them with patients with REM related OSAS as a useful way to evaluate the impact of a reduction of muscle hypotonia and respiratory variables in both groups. In our study, the nadir SpO_2_ of patients OSAS RBD achieves a lower drop of the nadir SpO_2_ compared to those observed in OSAS REM patients. The improvement observed in RBD on OSAS severity, denoted by a lower drop nadir SpO_2_, was named by us “the respiratory RSWA benefit”. This finding agrees with what was recently observed in patients with PD and OSAS with or without RBD^21^ and also observed in patients with OSAS RBD and OSAS controls^14^. These findings suggested that a shorter duration for sleep events and a consequent minor reduction of nadir oxygen saturation were related to an enhanced of active Pcrit (active critical closing pressure of the upper airway) in OSAS RBD patients.

Further studies regarding the mechanistic of neuromuscular control and Pcrit of the upper airway should be done to address this point. Another observation reported a more profound respiratory alteration in patients with PD with RBD^16^, which would mean the loss of the “respiratory sleep benefit” here described. Although the cause for this defeat is uncertain, it could be related to more advanced stages of the disease that involved brainstem neural structures regulating neuromuscular control of the upper airway in PD^24^. A future task will be to stratify respiratory polysomnographic findings according to the evolutionary stage of patients with PD.

Several limitations should be noted when interpreting our results. First, the sample size is relatively small. Second, despite there was no significant differences in the male-female ratio, this ratio tended to be higher in the OSAS RBD group. Third, the AH index was slightly lower, although not statistically significant in the OSAS REM group. Fourth, given that the data were collected from a single night we cannot exclude night-to-night variability. Similar limitations are founded in different studies that used similar research strategies. These limitations may be related to the low prevalence of this disorder and the bias in the selection of patients, coming from the clinic or the general population^25^. Despite these limitations, it is worth to note that, as far we know, this is the first study demonstrating that RBD have a “respiratory RSWA benefit” by alleviating OSAS severity in comparison to OSAS REM patients. Could this group integrated by the OSAS REM patients become a natural model to compare the “respiratory RSWA benefit” observed in patients with RBD and in some reports of PD^15^^,^^16^? This is a question that arises in the face of evidence from different drugs, particularly, cannabinoids, which reduce sleep apnea and RBD clinical features. We are aware that these preliminary findings require further research before to be recommended. However, they point out to a refreshing new alternative to standard treatments such as CPAP and also, in the case of RBD, by ameliorating the clinical manifestations related to the violent dreams enacting and/or coexisting OSAS RBD. From a pharmacological point of view, patients with OSAS REM could be useful to compare respiratory variables in the search for putative beneficial effects related to different therapeutic strategies in patients with concomitant OSAS RBD^25^^-^^26^.
